# Milling Surface Roughness Prediction Based on Physics-Informed Machine Learning

**DOI:** 10.3390/s23104969

**Published:** 2023-05-22

**Authors:** Shi Zeng, Dechang Pi

**Affiliations:** College of Computer Science and Technology, Nanjing University of Aeronautics and Astronautics, Nanjing 210016, China

**Keywords:** physics-informed deep learning, mechanism model, physically guided loss function, bi-directional gated recurrent unit

## Abstract

Surface roughness is a key indicator of the quality of mechanical products, which can precisely portray the fatigue strength, wear resistance, surface hardness and other properties of the products. The convergence of current machine-learning-based surface roughness prediction methods to local minima may lead to poor model generalization or results that violate existing physical laws. Therefore, this paper combined physical knowledge with deep learning to propose a physics-informed deep learning method (PIDL) for milling surface roughness predictions under the constraints of physical laws. This method introduced physical knowledge in the input phase and training phase of deep learning. Data augmentation was performed on the limited experimental data by constructing surface roughness mechanism models with tolerable accuracy prior to training. In the training, a physically guided loss function was constructed to guide the training process of the model with physical knowledge. Considering the excellent feature extraction capability of convolutional neural networks (CNNs) and gated recurrent units (GRUs) in the spatial and temporal scales, a CNN–GRU model was adopted as the main model for milling surface roughness predictions. Meanwhile, a bi-directional gated recurrent unit and a multi-headed self-attentive mechanism were introduced to enhance data correlation. In this paper, surface roughness prediction experiments were conducted on the open-source datasets S45C and GAMHE 5.0. In comparison with the results of state-of-the-art methods, the proposed model has the highest prediction accuracy on both datasets, and the mean absolute percentage error on the test set was reduced by 3.029% on average compared to the best comparison method. Physical-model-guided machine learning prediction methods may be a future pathway for machine learning evolution.

## 1. Introduction

Advanced machining technology is highly integrated with the latest achievements in modern science and technology. The current demand for high efficiency and quality in the machining industry has made high precision and high speed the core goals. Milling is widely used as an advanced machining technology in machining processes due to its high efficiency and productivity [1,2]. To ensure the serviceability of the ultimate mechanical product, rigorous control of the quality of the machined workpiece is crucial [3]. In recent years, researchers have conducted numerous studies related to surface quality in the milling process, during which numerous breakthroughs have been achieved [4,5,6]. Surface roughness often serves as an important indicator of surface quality in milling operations [7] and is directly used to monitor workpiece mechanical properties such as durability, surface friction and fracture resistance. For improving the practical utility of manufactured products, it is necessary to accurately quantify the surface roughness, and surface roughness prediction is gradually becoming a hot spot of theoretical and applied research in CNC machining [8]. The machining industry demands an increased surface quality while reducing manufacturing costs [9,10], so quantifying surface roughness is critical to parameter selection and has attracted many researchers and engineers to work on it [11].

The quantification of surface roughness during milling can be broadly divided into direct methods based on measurement and indirect methods based on modeling [12,13]. Direct methods based on measurements can be divided into contact and non-contact measurements. Contact measurement methods typically use contact-based stylus devices that have proven to be highly reliable for recording changes in the profile of a machined surface and thus deriving surface roughness. However, such methods have several limitations, such as long measurement times, additional requirements for certain environments and complex setups. In addition, these methods are not suitable for soft materials due to surface scuffing involved in the measurement process. To overcome the limitations of contact measurement, researchers proposed non-contact alternative methods based on machine vision. Compared to contact measurement methods, these methods have advantages such as a high measurement efficiency, easy setup and a non-invasive mode. However, shortcomings in prediction speed, prediction accuracy and consistency have hindered its large-scale diffusion in practical industrial applications.

To address a series of problems associated with direct methods based on measurements, researchers proposed indirect methods based on modeling to predict surface roughness. Indirect methods based on modeling can be broadly classified as physical and data driven. Physical models typically focus on mining the explicit physical relationships between surface roughness and the relevant input parameters, including the process parameters, tool vibration and the geometrical characteristics of the tool. Zhuo et al. [14] proposed a novel surface roughness prediction model considering cutting vibrations and the material removal rate. Compared to conventional surface roughness prediction models, this model considered the material removal rate, relative vibrations and the workpiece surface geometry between the tool and workpiece. Wang et al. [15] proposed a milling surface roughness prediction model based on geometric and mechanical factors related to surface roughness. The model considered not only elastic–plastic deformation, but also the effects of the tool parameters, the microhardness, the cutting forces and the material properties. Zhou et al. [16] analyzed the effect of cutting parameters, including the cutting speed, the feed rate and the lead angle, on the surface roughness based on the tool position and the workpiece surface geometry, and predicted the milling surface roughness. Although physical models have been a major success in surface roughness modeling, they have been difficult to apply in practical manufacturing due to the numerous uncontrollable complications in the milling process. In addition, the physical model predictive precision is closely associated with the domain knowledge of the practitioner and lacks practicality in the actual manufacturing industry.

With the rapid development of data science and computing capability, data-driven models have gradually entered the limelight. Data-driven methods employ statistical and machine learning models for end-to-end surface roughness predictions, enabling highly accurate predictions in scenarios without expert domain knowledge. Huang et al. [17] constructed a back-propagation neural network prediction method for forecasting roughness by using the signals collected by computerized CNC turning and sensing techniques as the input to the prediction model. Liu et al. [18] proposed a digital-twin-driven surface roughness prediction method to meet the real-time predictability requirements of smart manufacturing. Manjunath et al. [19] applied a long short-term memory network in surface roughness prediction on a S45C steel milling dataset. Du et al. [20] performed simulation modeling of the milling surface roughness by considering the realistic cutting edge track with miniature end mill dimensions, associated vibrations and material flexibility recovery. Wang et al. [21] proposed a roughness prediction method that takes into account tool wear variation in the case of variable cutting parameters. This methodology presented a roughness prediction model combining a stacked auto-encoder and a long short-term memory network in order to enable real-time monitoring of the surface quality of high value-added products, taking sensor signals and cutter wear conditions as inputs. However, the convergence of the above purely data-driven model to a local minima may lead to poor model generalization or results that violate existing physical laws. Therefore, there is an urgent necessity to integrate domain knowledge into relevant machine learning models that provide strong theoretical constraints.

In this paper, a novel prediction model for milling surface roughness was proposed by combining physical knowledge with deep learning. This study incorporates the physical mechanism model into the deep learning model in contrast to the existing work in this direction, which enables deep learning to perform surface roughness prediction under the constraints of physical laws. The embedding of physical knowledge enhances the generalization ability and prediction accuracy, providing a new method for surface roughness prediction. This model introduced the physical model in two phases of deep learning: before training and during training. Before training, a milling surface roughness mechanism model with tolerable accuracy was constructed to perform data augmentation on the limited experimental data. Data augmentation based on physical models not only expands the size of the input data, but also introduces the embedded mechanistic knowledge into the prediction model, subjecting it to potential mechanistic constraints. The proposed model guides the training process through the physically guided loss functions to take advantage of physical knowledge during training. This guidance mechanism enables the learning route of the model to converge to the region in the function space that conforms to the physical rules, improving the training efficiency and prediction rationality. Considering the excellent feature extraction capability of CNNs and GRUs in spatial and temporal dimensions, a CNN-GRU model was adopted as the main prediction model for milling surface roughness. In addition, a bidirectional gated recurrent unit model was introduced to combine the acquired forward and reverse output features into an output feature map for enhancing the correlation between the sequence data. Meanwhile, a multi-headed self-attention mechanism was introduced to aggregate the dimensional weights of the extracted features in the bidirectional gated recurrent unit layer to enhance the correlation between the data. The main contributions of this paper are as follows:

(1) The novel surface roughness prediction model integrates physical knowledge with deep learning to effectively solve the problem of data scarcity. In a limited data scenario, the proposed model has more accuracy and interpretability of prediction results than current methods.
(2) The loss function guided by the physical model constructed in the deep learning model is able to use existing mechanistic knowledge to train the model and guide the model to converge the learning route to the region in the function space that conforms to the physical rules, which effectively improves the training efficiency of the model.
(3) Physical knowledge was introduced as an input into the training process of the deep learning model to guide the learning process, resulting in a significant improvement in the accuracy of milling surface roughness predictions.

## 2. Related Work

### 2.1. Surface Roughness Prediction

The prediction of surface roughness during milling is critical to the design and manufacturing process of machine tools and has attracted many researchers to work on it. Surface roughness prediction methods can be broadly classified into physical models and data-driven models. Physical models typically focus on mining the explicit physical relationships between surface roughness and input parameters, including process parameters, tool geometry characteristics and tool vibration. Zhuo et al. [14] developed a novel surface topography prediction model considering cutting vibrations and material removal effects by simultaneously considering the relative tool–workpiece vibrations, material removal effects and the workpiece surface geometry. Wang et al. [22] investigated the effect of different lubrication methods on the surface roughness of TC21 titanium alloy during milling and developed a surface roughness model considering milling vibrations. Xu et al. [23] explored the dynamic displaceability of milling and the mechanism of roughness formation, proposing a related roughness prediction method based on the dynamic characteristics of milling. Wang et al. [15] proposed a milling surface roughness prediction model that simultaneously considered multiple factors such as the elastic–plastic deformation, the tool parameters, microhardness, the cutting force and the material properties based on the geometric and mechanical properties associated with surface roughness. Zhou et al. [16] explored the association of the cutting parameters, tool position and workpiece surface geometry with roughness and proposed a surface roughness prediction model. Lyu et al. [24] developed an analysis method for the formation mechanism of the roughness of the machined side of a straight groove end mill and proposed a numerical prediction model for the roughness in such a scenario.

With the rapid development of data science and computing capability, data-driven models have gradually entered the limelight. Data-driven methods employ statistical and machine learning models for end-to-end surface roughness predictions, enabling accurate predictions without domain experts. Kashyzadeh et al. [25] developed an artificial neural network based on a back-propagation error algorithm to predict the surface roughness of aluminum alloys under different machining conditions. Liu et al. [18] explored data-based tool wear and other roughness prediction techniques to predict roughness and an proposed an adaptive optimization method for process parameters using a digital twin. Huang et al. [17] developed a back-propagation neural network prediction model for surface roughness predictions in a multi-material scenario by collecting signals as input to the prediction model through CNC and sensing techniques during the machining process. Chen et al. [26] proposed a surface roughness prediction model based on a back-propagation neural network for machined workpieces, which could improve product quality and reduce machining costs simultaneously. Manjunath et al. [19] predicted the surface roughness during milling of S45C steel with a long short-term memory network, showing the advantages of the LSTM model in surface roughness predictions. Dubey et al. [27] considered the cutting speed, depth of cut, feed rate and nanoparticle concentration as input parameters and proposed a novel method to predict surface roughness with machine learning by varying the particle size of the cutting fluid to reduce time and energy wastage. Wu et al. [28] proposed a physics-informed surface roughness prediction model for milling machining, which effectively reduced the complexity and data dependence. Wang et al. [21] explored the correlation between tool wear and sensor signals with roughness and proposed a roughness prediction method with a hybrid self-encoder and a long short-term memory network.

### 2.2. Physics-Informed Machine Learning

In the current field of data science, the ability to collect and create observational data far exceeds the ability to understand data. Although purely data-driven models are suitable for data observation, observation bias will lead to poor model generalization and unreliable results. Therefore, it is urgent to integrate physical knowledge into relevant machine learning models to provide strong theoretical constraints. Physics-informed machine learning (PIML) enhances the capabilities of machine learning through building relevant models on the basis of empirical data and existing physical knowledge.

Typical supervised learning tasks such as classification and prediction are widely deployed in numerous application scenarios such as computer vision, time series analyses and multi-intelligent-body systems. Although deep learning methods have been the main solution to domain problems in recent years, there has been little integration of physical knowledge into spatio-temporal data tasks. Spatio-temporal data are data with both temporal and spatial dimensions and can be generated from irregularly spaced sensor networks in areas such as traffic [29], weather [30] and electricity [31]. Considering the consistency of the process of spatio-temporal data generation with physical laws, the physics-based approach is feasible in neural network model optimization. Guen et al. [32] clearly separated PDE dynamics from unknown complementary information through a two-branch depth architecture. The performance of video prediction was effectively improved by performing PDE constraint prediction in the latent space, inspired by data assimilation techniques. He et al. [33] proposed a physics-inspired deep learning approach for point cloud processing driven by natural flow phenomena. This method applied a static background grid to the joint data definition in the Eulerian world space and the Lagrangian material space with moving particles. The above representation enabled the natural evolution and accumulation of particle features using the flow rates generated by the generalized high-dimensional force fields. Finally, the effectiveness of the proposed model on various point cloud classification and segmentation problems was demonstrated by an excellent performance. Seo et al. [34] proposed physics-aware differential graph networks to learn finite differences inspired by physics equations using adjacent information. This architecture exploited data-driven end-to-end learning to discover potential dynamic relationships between spatial and temporal differences in a given continuous observation, offering significant advantages in prediction tasks for both synthetic and real climate observations. Iakovlev et al. [35] proposed a general continuous time differential model for dynamical systems. The control equations of the model were parameterized by a message-passing graph neural network. This model allowed for arbitrary spatial and temporal discretization, enabling effective neural partial differential inference while eliminating restrictions on observation point locations and time intervals.

## 3. Surface Roughness Prediction Model Based on Physics-Informed Deep Learning

**Figure 1:**
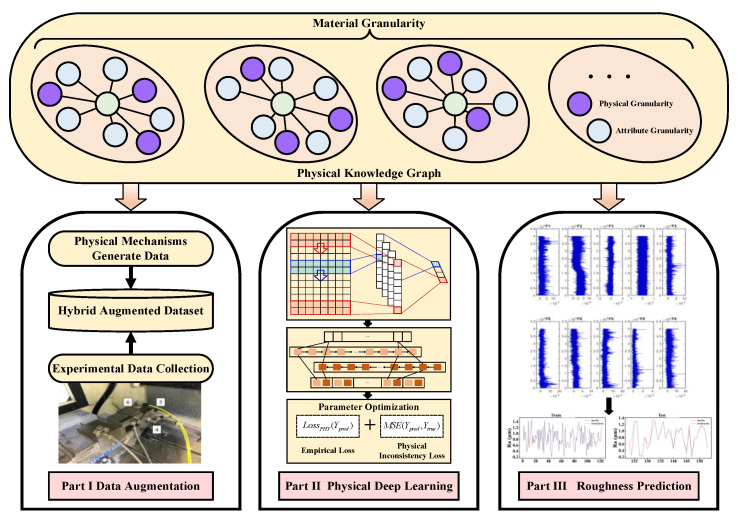
Structural diagram of the physically guided surface roughness prediction model.

The capability of data collection in the current data science field far exceeds the capability of data comprehension. The convergence of a purely data-driven model to local minima may lead to poor model generalization or results that violate existing physical laws. Therefore, it is urgent to integrate domain knowledge into relevant machine learning models to provide strong theoretical constraints and inductive biases on the basis of observations. This paper combined physical knowledge with deep learning to propose a surface roughness prediction model based on physics-informed deep learning (PIDL), the structure of which is shown in Figure 1.

The structure of the proposed model can be divided into three main parts, which are data augmentation, physical deep learning and roughness prediction. Part I is mainly responsible for generating the data used throughout the model and performing data augmentation through physical mechanisms. Part II performs feature extraction and learning based on CNN–GRU, where physical constraints have been introduced through the physically guided constructed loss function. Part III feeds the milling features derived from Part II into the prediction model for surface roughness prediction. The interaction between the three parts can be summarized as a progression, where Part I provides the data containing the physical laws for Part II, and Part II provides the surface roughness prediction model under the constraints of the physics for Part III. The proposed model includes physical knowledge before and during training in deep learning. Before training, data enhancement was performed on limited experimental data by constructing a surface roughness mechanism model with a tolerable accuracy. During training, a physically guided loss function was constructed to guide the training process of the model with physical knowledge. Considering the excellent feature extraction capability of convolutional neural networks and gated recurrent units in the spatial and temporal dimensions, a CNN–GRU model was adopted as the main prediction model for surface roughness. Meanwhile, bidirectional gated recurrent units and a multi-headed self-attentive mechanism were introduced to enhance data correlation. The following section describes the sub-models and their components included in the physically guided deep prediction model in detail.

### 3.1. Surface Roughness Mechanism Model

**Figure 2:**
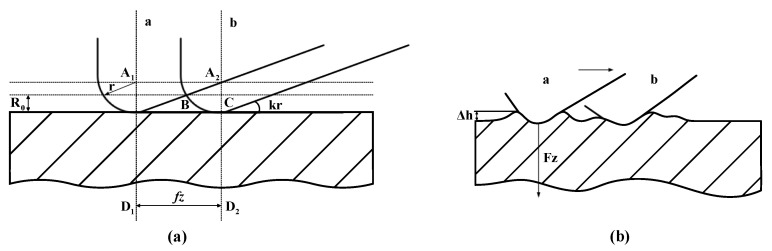
Diagram of the machining surface. (**a**) Simplified diagram of the formation of surface residual height. (**b**) Diagram of the cutting process.

During mechanical milling, the machined surface of the workpiece tends to not be completely flat. As an indicator to monitor the quality of workpiece machining, surface roughness is directly related to micro-geometry errors such as small spaces and micro-scale peak and valley inhomogeneities in the machining plane [36,37]. The relative motion between the workpiece and the tool during milling makes it impossible to completely eliminate the theoretical cutting area in actual machining. As shown in Figure 2, many areas of the workpiece surface are left after the actual milling process, which are referred to as residual areas. The value of the theoretical roughness is directly related to the maximum residual height of the residual area.

The physical modeling of surface roughness was carried out by combining geometric and mechanical models for the residual areas of the machined surfaces mentioned above. The specific flow of this physical modeling process is referred to in [15], and this paper demonstrates a simplified physical modeling process. Assuming that the surface roughness of the machined workpiece is only related to the height of the residual area in the actual machining process, the theoretical expression for the arithmetic contour mean is shown in Equation (1).

(1) Ra=CRz=C(R0+∆h)

where C denotes the proportional relationship between Ra and Rz and R0 denotes the maximum residual height of the machined residual region. The mechanism of the formation of residual height of the machined surface during the movement of the tool from position *a* to *b* is visualized in Figure 2a. The three points $A_{2}$, B and C in the diagram are the intersection points between the marked horizontal dashed line and the solid line of the tool, which constructs a right triangle $A_{2}\mathrm{CB}$. The following theoretical relationships can be obtained from the labeled geometric relationships in the diagram, as shown in Equations (2) and (3).

(2) $${\mathrm{BA}}_{2}{}^{2}={\mathrm{BC}}^{2}+{\mathrm{CA}}_{2}{}^{2}$$

(3) $$r^{2}={\left(fz-R0\cot kr\right)}^{2}+{\left(r-R0\right)}^{2}$$

where kr denotes the auxiliary angle of the tool, r denotes the radius of the arc of the tool tip and fz denotes the feed per tooth. The theoretical expression for the maximum residual height, R0, can be obtained by simplifying the above equation, as shown in Equation (4).

(4) $$R0=\frac{1}{2}fz\sin2kr+r\sin kr^{2}-\sin^{2}kr\sqrt{r^{2}-fz^{2}+2rfz\cot kr}$$

In the actual machining process, the surface roughness of the workpiece is related to geometric factors and the mechanical properties of the material. The extrusion phenomenon of the tool on the surface of the workpiece during the cutting process results in elastic–plastic deformations of the machined surface. In view of the above influence of the mechanical properties on surface roughness, a corresponding physical model is constructed for the residual height, ∆h, of the workpiece surface. The geometric meaning of the residual height, ∆h, is shown in Figure 2b, which consists of the plastic deformation height and elastic rebound, and its theoretical expression is shown in Equation (5).

(5) ∆h=h−δ

The plastic deformation height, h, can be obtained by the Krakelsk principle of frictional wear calculations, the mathematical expression of which is shown in Equations (6) and (7).

(6) $$\sigma x=\frac{\sigma }{1-\psi }=\frac{0.33Hv}{1-\psi }$$

(7) $$\psi =\frac{h}{2r}$$

where r denotes the micro-bump radius, i.e., the radius of the arc of the tool tip during cutting, Hv denotes the Vickers hardness of the material and σ denotes the flow stress. After simplifying the above equation, the plastic deformation height, h, can be expressed in Equation (8).

(8) $$h=2r\left(1-\frac{0.33Hv}{\sigma }\right)$$

where the flow stress σ can be obtained by the Johnson–Cook principal structure equation, the mathematical expression of which is shown in Equation (9).

(9) $$\sigma =\left(A+B\varepsilon^{n}\right)\left(1+C_{0}\left(\frac{\varepsilon 1}{\varepsilon 0}\right)\right)(1-{\left(\frac{T-T\infty }{Tm-T\infty }\right)}^{m})$$

where A, B and $C_{0}$ denote the equation coefficients, ε denotes the strain rate; ε1 and ε0 denote the equivalent strain rate and the reference strain rate, respectively, T denotes the workpiece temperature, T∞ denotes room temperature and Tm denotes the melting temperature of the workpiece. Besides the plastic deformation height h, the elastic rebound, δ, in the remaining height, ∆h, can be calculated by Hertz elastic contact theory, the mathematical expression of which is shown in Equations (10)–(12).

(10) $$\delta =\left(\frac{1-v1^{2}}{E1}+\frac{1-v2^{2}}{E2}\right)\frac{\pi aqmax}{2}$$

(11) $$q\max=\frac{3F}{2\pi a^{2}}$$

(12) $$a=\sqrt[{3}]{\frac{3FR1R2}{4(R1+R2)}\left(\frac{1-v1^{2}}{E1}+\frac{1-v2^{2}}{E2}\right)}$$

(13) $$\delta =\left(\frac{1-v1^{2}}{E1}+\frac{1-v2^{2}}{E2}\right)\cdot \sqrt[{3}]{\frac{9F^{2}}{16R1}}$$

where F denotes the *z*-axis cutting force during cutting, R1 and R2 denote the radii of the two contact bodies, respectively, E1 and E2 denote the modulus of elasticity of the tool and the machining material, respectively, and v1 and v2 denote the Poisson’s ratio of the tool and the machining material, respectively. On this basis, the elastic rebound, δ, in the remaining height, ∆h, can be calculated by Equation (13).

### 3.2. Physically Guided Deep Prediction Model

Deep learning has been demonstrated to be robust in terms of feature extraction, time series prediction, etc. This paper combines physical knowledge with deep learning to propose a physically guided surface roughness prediction model. The proposed prediction model consists of physical knowledge and neural networks. Considering the convincing feature extraction capability of CNNs for time-series data, this paper adopted a CNN for milling feature extraction to track the spatial pattern variations caused by process parameter differences. The CNN can feed pre-processed data directly into the model and extract the deep non-linear features hidden in it by combining multiple convolution and pooling layers. Since GRUs are capable of learning sequential and time-varying patterns from the original dataset, this paper employed a GRU for the dynamic tracking of surface changes during the tool life cycle. Compared with the LSTM architecture, GRUs have the advantages of faster convergence and a fewer number of parameters. This paper adopted a hybrid deep learning model, CNN–GRU, as the neural network part of the prediction model. Physical knowledge was mainly integrated in the training phase and the loss function part in order to use the physical knowledge to guide the training process of the model.

**Figure 3:**
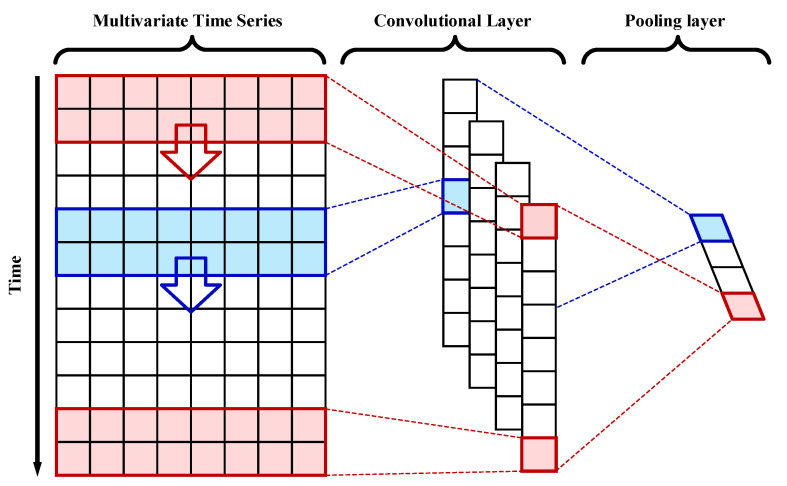
The specific network structure of a 1D-CNN, where the colors indicate different filters.

CNNs are one of the most successful deep learning methods, and their network structure can be divided into 1D-CNNs, 2D-CNNs and 3D-CNNs. A 1D-CNN is very suitable for time series analyses of sensor data or analyses of periodic signal data, and its specific structure is shown in Figure 3.

The CNN architecture typically consists of a convolutional layer and a pooling layer to filter and extract useful features from the input data. The leftmost part of the figure shows the input multi-dimensional time series data. Each convolutional layer has a corresponding convolutional kernel, and each colored box on the input data in the figure represents a filter. The filter slides top-down over the entire input matrix, producing convolutional features of the input data through a contained coefficient matrix. The dimension of the convolutional features extracted by the filter is N∗1, where N is related to the input dimension, the filter size and the convolutional step size. Assuming that the number of convolutional kernels applied to the input data is *M*, then the dimension of the extracted convolutional features is N∗M. The convolution layer tends to be followed by a nonlinear activation function and then this is immediately followed by a pooling layer. The pooling layer is a subsampling technique that can transform and aggregate each convolutional feature matrix into a low-dimensional feature matrix based on specific rules. For example, the maximum value in the current sliding window, i.e., the most critical feature in the window, will be output under the max-pooling rule. Pooling operations can enhance the robustness of the system and reduce the sensitivity of the pooled output to changes in the input. In summary, the CNN architecture is suitable for extracting robust features of time series data and avoiding the iterative expansion of matrix dimensions.

**Figure 4:**
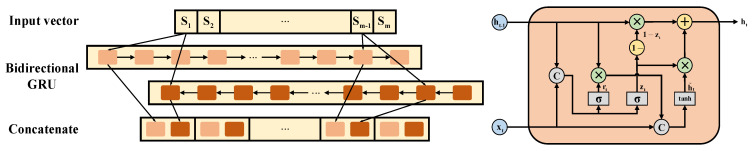
Bidirectional GRU network structure and detailed structure of GRU units.

Recurrent neural networks (RNNs) are capable of memorizing historical data features and are applicable to a variety of sequential data problems. Gated recurrent unit (GRU) neural networks selectively filter and remember historical information through update gates and reset gates, solving the problems of gradient disappearance, gradient explosion and poor long-term memory of traditional RNNs. Due to the temporal characteristics of vibrational data, this paper employs a GRU to extract vibration sequence features. The detailed internal structure of the GRU model is shown in Figure 4, where $h_{t-1}$ and $h_{t}$ represent the hidden states of the previous unit and the current unit, respectively, ${\tilde{h}}_{t}$ represents the candidate state of the current unit and $x_{t}$ represents the input tensor of the current unit. Specifically, $h_{t}$ and ${\tilde{h}}_{t}$ are obtained through $h_{t-1}$ and $x_{t}$, which are calculated as shown in Equations (14)–(17).

(14) $$r_{t}=\sigma \left(W_{r}\cdot \left[h_{t-1};x_{t}\right]\right)$$

(15) $$z_{t}=\sigma \left(W_{z}\cdot \left[h_{t-1};x_{t}\right]\right)$$

(16) $${\tilde{h}}_{t}=\tanh\left(W_{h}\cdot \left[r_{t}⊙h_{t-1};x_{t}\right]\right)$$

(17) $$h_{t}=h_{t-1}⊙\left(1-z_{t}\right)+{\tilde{h}}_{t}$$

where $r_{t}$ and $z_{t}$ represent the reset gate and update gate, $W_{r}$, $W_{z}$ and $W_{h}$ represent weight matrices, $h_{t}$ consists of historical and current state information, $h_{t-1}⊙\left(1-z_{t}\right)$ represents the selective forgetting of historical information and ${\tilde{h}}_{t}⊙Z_{t}$ represents the selective memory of current information. While processing vibration data with a GRU, the clarity of the historical information inherited from previous units is negatively correlated with the length of the sequence. To enhance the correlation between the dimensions of the time-series vibration data, a bidirectional GRU and a multi-headed self-attention mechanism were introduced. The timing vibration data are fed into the bidirectional GRU from the forward and reverse directions, respectively, where the specific structure of the network and cells is shown in Figure 4. The bidirectional GRU combines the acquired forward and reverse output features into an output feature map, which enhances the correlation between the dimensions of the time-series vibration data.

**Figure 5:**
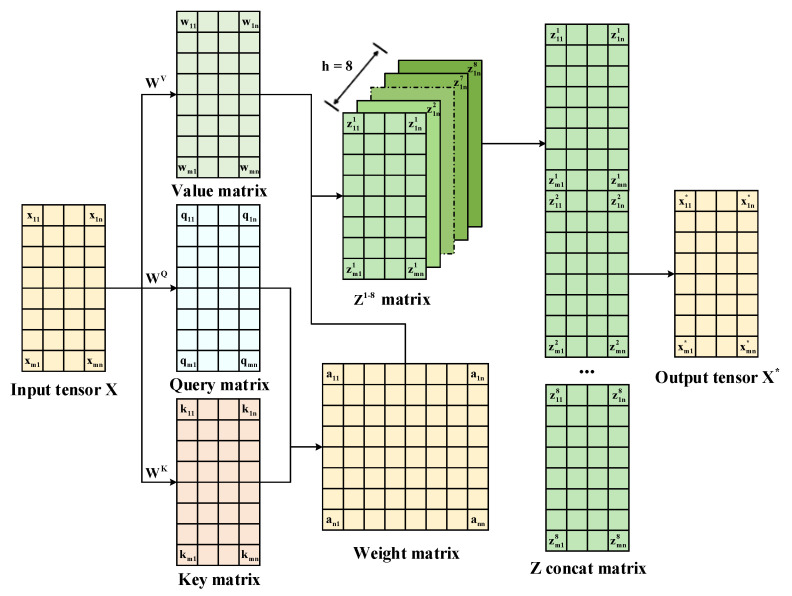
Multi-headed self-attentive mechanism structure.

A multi-headed self-attention mechanism can enhance the correlation between dimensions of sequence data, and is widely used in the fields of natural language processing and image processing. This paper set the head number of the multi-headed self-attentive mechanism to eight, and its specific structure is shown in Figure 5.

The output feature mapping of the bidirectional GRU served as its input tensor, and the three feature matrices Query, Key and Value were calculated with $W^{K}$, $W^{Q}$ and $W^{V}$, respectively. The correlation between the Query matrix and the Key matrix was characterized by the dot product operation, and the calculation procedure is shown in Equation (18). Then, the calculated result was divided by the column dimensions of the Query matrix to avoid the occurrence of oversized results that are difficult to observe. Finally, the above result was converted into a weight matrix by the softmax operation, and the Z matrix was obtained by multiplying the weight matrix with the Value matrix.

(18) $$Z=\mathrm{softmax}\left(\frac{Q^{T}K}{\sqrt{d_{k}}}\right)V$$

The multiple Z matrices calculated above were executed and transformed into the target output tensor X*. The input tensor and the output tensor have the same data structure, and all dimensions of the output tensor take the relevance of the remaining dimensions into account. This mechanism can fully extract and enhance the correlation between the vibrational data dimensions.

The bidirectional GRU mainly consists of three GRU layers and a multi-headed self-attentive mechanism. In order to ensure a reasonable training time at the current model parameter scale, the first GRU layer is a bidirectional GRU layer that extracts vibrational data location features in both the forward and reverse dimensions. Then, a multi-headed self-attentive mechanism was placed after the bidirectional GRU layer for weight setting. This mechanism enables aggregating the dimensional weights of the extracted output features in the bidirectional GRU layer to enhance data correlation. Finally, the remaining GRU layers were set as normal GRU layers for extracting the features of the output matrix from the multi-headed self-attentive mechanism.

Physical knowledge was introduced in the training phase and loss function part of the above deep learning model to guide the training process of the model with physical knowledge. Compared to the conventional loss function, the proposed physically guided loss function was capable of capturing dynamic patterns that can be generalized in the framework of defined physical laws, and its mathematical model is shown in Equation (19).

(19) $$Loss=Loss_{TRN}(Y_{true},Y_{pred})+\lambda R(W)+\gamma Loss_{PHY}(Y_{pred})$$

where $Loss_{TRN}(Y_{true},Y_{pred})$ and λR(W) are the same loss factors as used in most machine learning models and $\gamma Loss_{PHY}(Y_{pred})$ denotes a loss factor based on physical knowledge, which ensures that the training learning process of the prediction model is consistent with physical laws.

The specific expression of physical knowledge normally depends on factors such as milling material, milling environment, static parameter settings, etc. Take S45C steel as an example. In the practical milling process, the surface roughness is positively correlated with the vibration frequency, i.e., the surface roughness increases with the increase in vibration frequency. The vibration frequency decreases as the spindle speed increases. When the feed speed falls within the range of [100,200] mm/min, the vibration frequency increases with the increase in feed speed, and when the feed speed exceeds 200 mm/min, the vibration frequency changes insignificantly with the increase in feed speed. As a result, the surface roughness of S45C steel decreases with the increasing spindle speed as well as the increasing feed rate. To enable the above physical laws to serve as a guide for the model training process, a positive and negative monotonicity loss function was constructed, the mathematical model of which is shown in Equations (20) and (21).

(20) $$Loss_{D_{1}}(f\left(t,x_{1}\right),f\left(t,x_{2}\right))=\overset{m}{\underset{i=1}{\sum }}((x_{1}^{i}<x_{2}^{i})\wedge (f\left(t,x_{1}^{i}\right)>f\left(t,x_{1}^{i}\right))\cdot \mathrm{ReLU}(f\left(t,x_{1}^{i}\right)-f\left(t,x_{2}^{i}\right)))$$

(21) $$Loss_{D_{2}}(f\left(t,x_{1}\right),f\left(t,x_{2}\right))=\overset{m}{\underset{i=1}{\sum }}((x_{1}^{i}>x_{2}^{i})\wedge (f\left(t,x_{1}^{i}\right)>f\left(t,x_{1}^{i}\right))\cdot \mathrm{ReLU}(f\left(t,x_{1}^{i}\right)-f\left(t,x_{2}^{i}\right)))$$

where $Loss_{D_{1}}(f\left(t,x_{1}\right),f\left(t,x_{2}\right))$ and $Loss_{D_{2}}(f\left(t,x_{1}\right),f\left(t,x_{2}\right))$ denote the domain loss under the physical monotonicity constraint and the logical operator AND (∧) is responsible for determining the monotonicity relationship between each feature and the surface roughness. The ReLU as a nonlinear function can help the neural network model learn the nonlinear relationship, which can better capture the nonlinear relationship in the data compared to the linear function when training the neural network, thus improving the performance of the model. In addition, the gradient can easily disappear when the sigmoid function is back propagated in deep neural networks. ReLU can enhance the sparsity of the network, reduce the interdependence between parameters and alleviate the overfitting problem to improve training efficiency. Therefore, ReLU was selected as the activation function in this paper. The mask mechanism was applied to the ReLU function to ensure that the training loss on the physical level was considered only when the predictions of the neural network model were contrary to physical laws. When the selected features are physically negatively correlated with the surface roughness, $Loss_{D_{2}}\left(f\left(t,x_{1}\right),f\left(t,x_{2}\right)\right)$ will be considered in the total model loss if the predicted data are positively correlated, and conversely, $Loss_{D_{1}}\left(f\left(t,x_{1}\right),f\left(t,x_{2}\right)\right)$ will be used to calculate the physically guided loss. The physically guided loss function can guide the model to converge the learning route towards the region in the function space that conforms to physical laws, which effectively improves the training efficiency and physical rationality of the model. The above loss function considers the case of violation of physical laws from two different directions of evolution. The predictions that conform to physical laws are filtered out using the corresponding masking mechanism, and only the corresponding offending terms are superimposed, effectively placing physical constraints on the deep learning model. In summary, the physically guided loss function constructed above is incorporated into the global loss function to form the final loss function under the physical law constraints. This loss function enables the learning route of the model to converge to the region in the function space that conforms to physical laws, which is expressed in Equation (22).

(22) $$Loss=Loss_{TRN}(Y_{true},Y_{pred})+\lambda R(W)+\gamma (Loss_{D_{1}}+Loss_{D_{2}})$$

## 4. Experiment

In this section, the proposed method was compared with existing methods on open datasets. Moreover, ablation experiments were conducted to demonstrate the effectiveness of each component of the physically guided surface roughness prediction method.

### 4.1. Datasets and Metrics

The experiments verify the effectiveness of the proposed method with two open-source milling datasets. The first dataset was the open-source S45C steel milling dataset from Chung Hsing University, Taiwan, China, which performed cutting operations on S45C steel with tungsten carbide milling cutters [38]. T. Wu and K. Lei carried out this work, and all milling was performed on a CNC machine. All vibrations and corresponding combinations of cutting parameters included in the experimental dataset can be obtained from https://drive.google.com/drive/folders/1M6dgDNYhHbp8iFmNXeV9HrL6x6AvSshx?usp=share_link, accessed on 3 January 2019. The tool specifications used in the experiments are shown in Table 1. The vibration acceleration during milling was measured by attaching an accelerometer (Wilcoxon Research 785A) to the spindle and vise.

**Figure 6:**
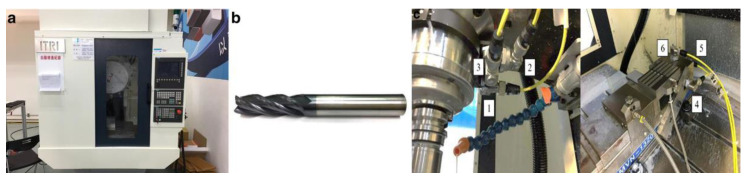
Experimental devices. (**a**) Picture of the milling machine used in this experiment. (**b**) Picture of the tungsten carbide milling cutter used in this experiment. (**c**) Picture of accelerometer installation on the spindle (1–3) and the vise (4–6).

In order to verify the proposed approach and evaluate the accuracy of predicting the workpiece surface roughness, a tungsten carbide milling cutter was utilized to cut the steel S45C in the experiment. Figure 6a,b shows pictures of the tooling machine and the cutter used in this experiment. As shown in Figure 6c, accelerometers (Wilcoxon Research 785A) were attached to the spindle and the vise to measure the vibration acceleration during the milling process.

A total of nine steel blocks with three different clamping torque straight cutting trajectories were used in the experiments. The cutting parameters include various spindle speeds, feed speeds, feed volumes, etc. The different combinations of cutting parameters and the expected material removal rate (MRR) of the cutter are shown in Table 2. The measured vibration signals were recorded by a data acquisition device (DAQ NI 9234) with a sampling rate of 10 KHz. Tool wear during milling is one of the factors that affects surface roughness. In this study, the limitations of instrumentation and quantification (e.g., estimation of wear edges by online microscopy) of tool wear detection prevented the accurate characterization of the cutter wear features. Each new cutter was only used in the milling process of one workpiece block (a total of nine cutting traces) in this experiment, and thus the estimated MRR was used to simply represent the factor of cutter wear.

**Figure 7:**
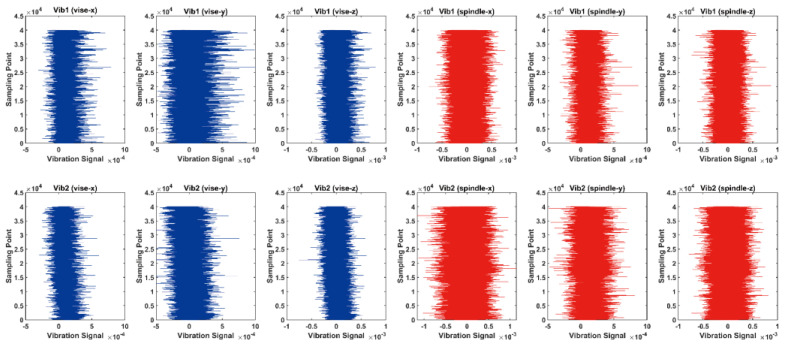
Vibration signals under different cutting parameters, where vise denotes the vise vibration, spindle denotes the spindle vibration and Vib1 and Vib2 represent two different sets of experiments.

A priori knowledge shows that the vibration signal is sensitive to tool wear and is therefore a good indicator of tool wear and surface roughness. The first and second rows in Figure 7 show the six vibration signals collected in two sets of milling experiments with different cutting parameters. These two sets of experiments were identical except for the cutting depth, which was set to 0.5 mm and 0.9 mm. Specifically, the spindle speed was set to 3000 rpm, the feed rate was 240 mm/min, the feed per tooth was equal to 0.02 mm/tooth and the clamping torque of the vise was equal to 18 N-m. It could be found that the amplitude of the vibration signals trended to increase as the cutting proceeded. For vise vibrations, the depth of cut is negatively correlated with the vibration amplitude, while the opposite is the case for spindle vibrations. Once the milling process was finished for one block of workpiece according to the different combinations of cutting parameters, the surface roughness of the workpiece was measured in terms of average roughness by a Mitutoyo SV-C3200S4 instrument, which is capable of measuring the surface profile of the workpiece within the range of 100 mm in the *x*-axis and 8/80/800 μm in the *y*-axis with the corresponding resolutions of 0.0001/0.001/0.01 μm.

The second dataset was the micromechanical milling dataset from the GAMHE 5.0 pilot line. The Workstation 4 (WS4) of the GAMHE 5.0 pilot line is a Kern Evo high precision machining center with a maximum spindle speed of 50,000 rpm, equipped with a Blum laser system for running micro-milling and micro-drilling operations. This dataset contains results from micro-milling operations on a sintered tungsten–copper alloy (W78Cu22) with 0.3 mm, 0.5 mm, 0.8 mm and 1 mm diameter milling cutters. Five cutting parameters were considered during the experimental machining process: spindle speed, feed rate, axial depth of cut, radial depth of cut and milling tool radius. The different combinations of cutting parameters are shown in Table 3. Meanwhile, data such as root mean square values and peak values of machine *x*, *y* and *z* axes vibrations during micro milling operation were collected. The monitoring system platform is equipped with a Deltatron 4519-003 Brüel and Kjaer monoaxial accelerometer on the *x*, *y*, *z*-axes, with a sensitivity of 10.58 mV/g and a bandwidth of 20 kHz. The sensor was connected to a PCB PZT 482A22 amplifier to supply power in order to obtain an acceptable voltage measurement which can be easily processed by most data acquisition systems. The vibration signals were fed into an NI 6251 National Instruments data acquisition card, with a sampling frequency of 50 kHz, and processed in a National Instruments high performance PXI 8187 embedded controller. Both of the above open-source datasets are allowed to be replicated, manipulated and used in other works, and since the same techniques and processes are reproducible in practical applications in manufacturing engineering, the above datasets were used for experiments in this study.

Three performance evaluation metrics, namely the coefficient of determination (R2), the root mean square error (RMSE) and the mean absolute percentage error (MAPE), were adopted to assess the performance of the proposed model. The root mean square error is a typical metric for regression models to indicate the extent of the error that the model will produce in its predictions, representing the sample standard deviation of the difference between the predicted and true values. The mean absolute percentage error is a relative error measure that avoids positive and negative errors canceling each other out through absolute values, and is commonly adopted to measure the accuracy of various time series prediction models. The coefficient of determination reflects how accurately the model fits the data and usually takes a value in the range [0–1]. The coefficient of determination is positively correlated with the explanation ability of the current equation variables of the target quantity. The computation of R2, RMSE and MAPE is shown in Equations (23)–(25), where n denotes the total number of samples, $y_{i}$ denotes the true value of the i-th sample, ${\hat{y}}_{i}$ denotes the predicted value of the *i*-th sample and $\bar{y}$ denotes the mean of the true values.

(23) $$R^{2}=1-\frac{\sum_{i=1}^{n}{\left(y_{i}-{\hat{y}}_{i}\right)}^{2}}{\sum_{i=1}^{n}{\left(y_{i}-\bar{y}\right)}^{2}}$$

(24) $$\mathrm{RMSE}=\sqrt{\frac{1}{n}\sum_{i=1}^{n}{\left(y_{i}-{\hat{y}}_{i}\right)}^{2}}$$

(25) $$\mathrm{MAPE}=\frac{100\%}{n}\sum_{i=1}^{n}\left|\frac{{\hat{y}}_{i}-y_{i}}{y_{i}}\right|$$

### 4.2. Baseline Methods

In this paper, the following models were used as for comparison to verify the validity of the proposed model:

(1) ANNs (Huang et al. [17]): This study employed artificial neural networks with a large number of interconnected neurons to search for a good fit of the root mean square error in training and validation. The best prediction of surface roughness was obtained through the development of back-propagation neural network prediction models in both single and multi-material models. This study input static milling features into an artificial neural network model for roughness prediction, neglecting the temporal and spatial data features.

(2) LSTM (Mahjoub et al. [39]): This study fed a power consumption time series dataset into an LSTM neural network model to predict future load consumption and avoid consumption spikes. The prediction model based on LSTM can provide ahead of time decisions and trigger load shedding in cases of overload. This study adopted the LSTM model for prediction, which considered the characteristics of the input data over the time span and ignored the spatial characteristics of the data and the embedded established physical rules.

(3) GRU (Liu et al. [40]): This study proposed a method for predicting satellite network traffic based on gated recurrent units. The method can fully exploit the time dependent features of the input sequences by a GRU neural network and mine the similar correlations between data sequences. This study is similar to the LSTM model, which ignores the spatial characteristics and physical properties of the data, although it considers the features in the temporal dimension.

(4) SAE–LSTM (Wang et al. [21]): This study proposed a surface roughness prediction method considering dynamic tool wear under variable cutting parameter scenarios. This method employed a hybrid prediction model based on stacked autoencoders and long short-term memory networks for high accuracy prediction from tool wear conditions and sensor signals. This study considered both the spatial and temporal features of the input sequence, ignoring the physical knowledge embedded in the sequence data.

(5) CNN–LSTM (Choudhury et al. [41]): This study proposed a CNN–LSTM model with an adaptive batch size to recognize various types of human activities in uncontrollable environments. This model was iteratively trained and validated using adaptive batch sizes ranging from 128 to 1024. This study extracted features of spatial and temporal dimensions from the input data through a CNN and an LSTM, respectively, ignoring the vital guidance of the physical knowledge embedded in the sequence data.

(6) CNN–BGRU (Shang et al. [42]): This study proposed an automatic prediction technique for the remaining useful life (RUL) of rolling bearings based on deep learning networks. Firstly, spatial features were learned from the bearing condition monitoring data by convolutional neural networks. Then, a bidirectional GRU was used to extract the time-degrading trend from the data. Finally, the weighted average method was employed to smooth the trend of RUL prediction, which provided more accurate results for bearing RUL prediction. This study separately performed feature extraction from a spatio-temporal perspective, but ignored the physical knowledge embedded in the data, reducing the usefulness of the model.

### 4.3. Experimental Results and Analysis

**Figure 8:**
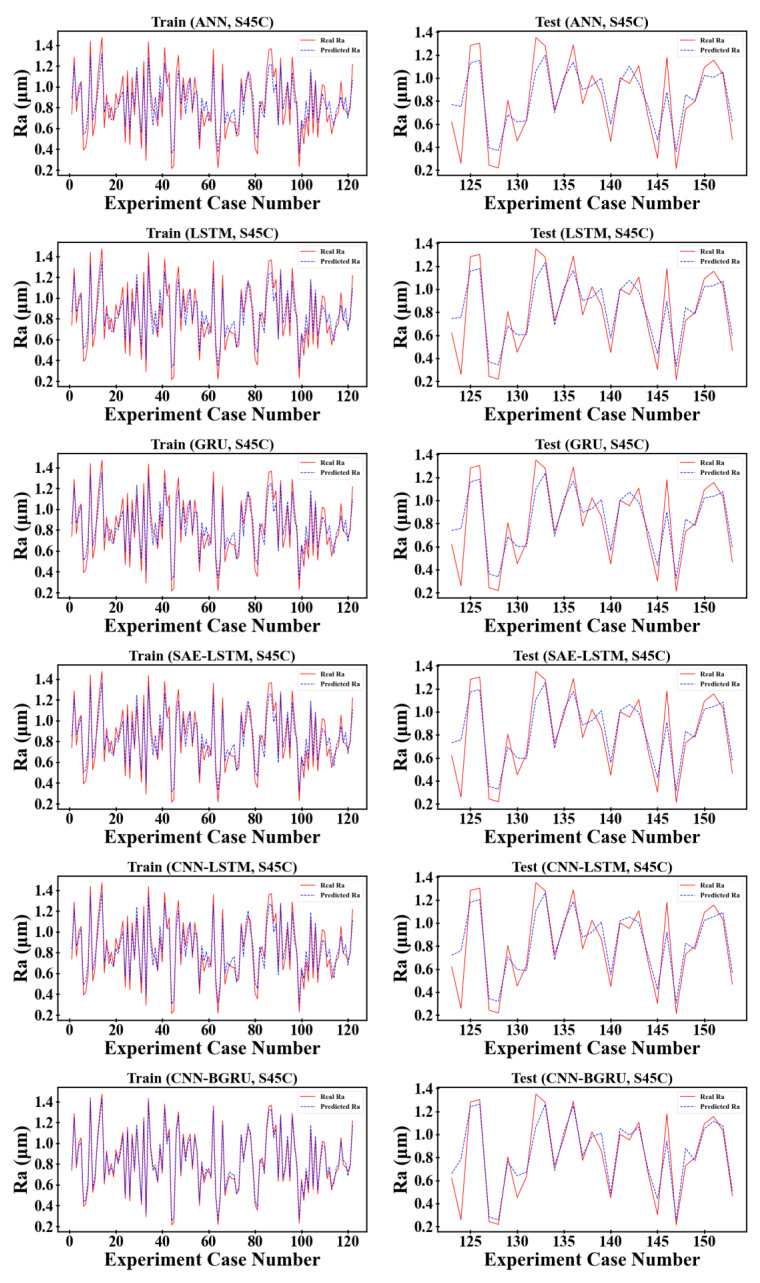

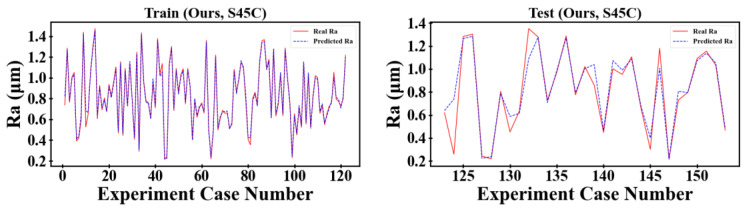
Prediction results of different methods on the S45C dataset.

**Figure 9:**
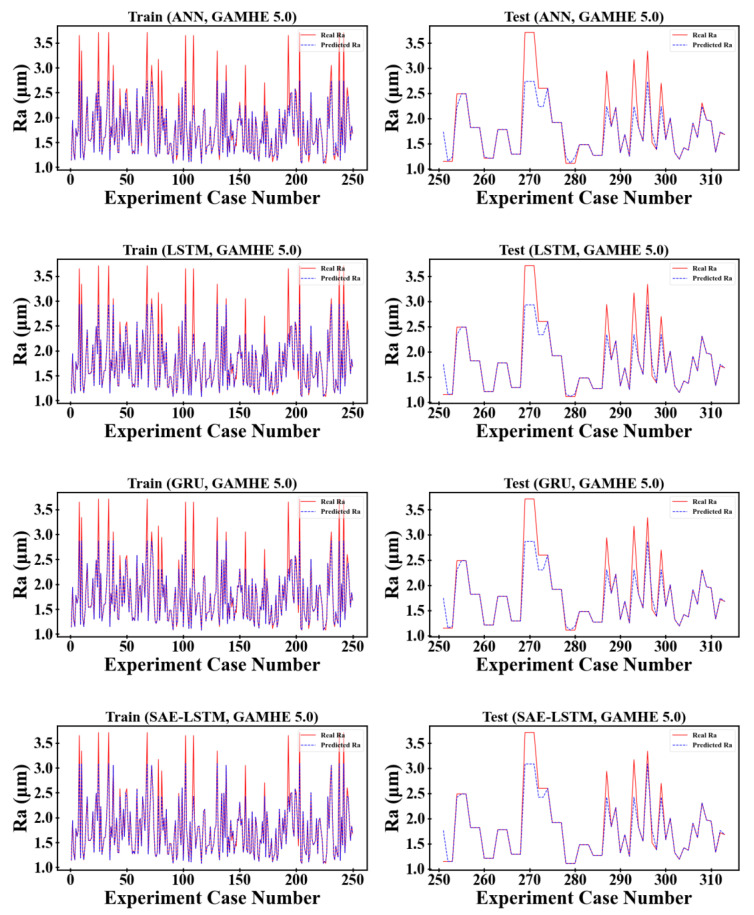

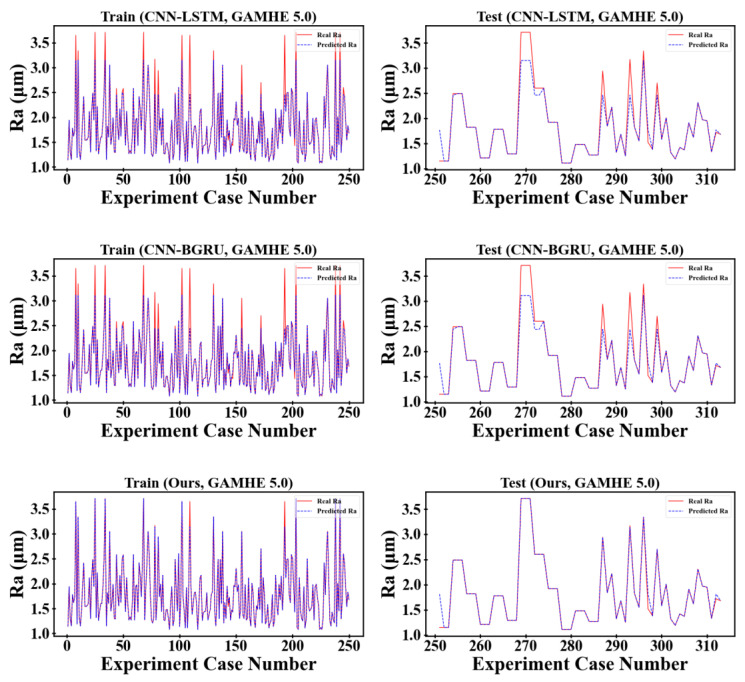
Prediction results of different methods on the GAMHE 5.0 dataset.

The proposed surface roughness prediction model was validated on the open-source S45C steel milling dataset and the micromechanical milling dataset in the GAMHE 5.0 test line and compared with classical and state-of-the-art methods proposed in recent years. Considering the generalization and persuasiveness of the two dataset divisions, we selected 80% of the dataset as the training set and 20% as the test set based on 10-fold cross-validation to fully demonstrate the performance of the proposed model. The comparison results of the evaluation metrics for each prediction method are shown in Table 4 and Table 5, and the prediction results of each prediction method on the training and test sets are shown in Figure 8 and Figure 9.

As can be seen from Table 4 and Table 5, the surface roughness prediction model based on physics-informed machine learning proposed in this paper outperforms the other comparison models on both the S45C steel milling dataset and the GAMHE 5.0 micromachining milling dataset. The S45C steel milling dataset is smaller in data size than the GAMHE 5.0 micromachining milling dataset, so the prediction accuracy of the overall model is reduced. In the S45C steel milling dataset, the overall effect of the LSTM is slightly less than that of the GRU. As the data size increases, the prediction accuracy of the LSTM is better than that of the GRU for the GAMHE 5.0 micromachining milling dataset. The CNN–LSTM model overall outperforms SAE–LSTM on both datasets, indicating that the convolutional neural network possesses stronger feature extraction capability than the stacked autoencoder. In both the training and testing phases of the S45C steel milling dataset, the prediction accuracy of the CNN–BGRU model outperformed that of the CNN–LSTM, indicating that the bidirectional gated recurrent units can improve efficiency while learning the patterns and connections embedded in the time series data more comprehensively from both forward and reverse sequences. The RMSE and MAPE values increase from the training phase to the testing phase, while the opposite occurs for R2. Both the RMSE and the MAPE are negatively correlated with prediction accuracy, and a value of 0 indicates that the predicted and real values exactly match. In contrast, the R2 metric is positively correlated with the prediction accuracy and indicates perfect prediction when taking a value of 1. Therefore, the phenomena of RMSE and MAPE becoming larger and R2 becoming smaller from the training phase to the testing phase indicate that the model may have been underfitted or overfitted. This is mostly due to weak model generalization due to the insufficient number of training samples. The mean absolute percentage error of the proposed model on the two datasets drops by 4.46% and 1.598% compared to the best comparison models CNN–BGRU and CNN–LSTM.

In the following, the experimental results of each prediction model on the training and testing sets are combined to analyze the reasons for the improvement presented by the proposed model.

(1) In this paper, a model of the physical mechanisms of milling surface roughness was constructed by combining geometric and mechanical models of the residual area of the machined surface. The data augmentation of the milling experimental dataset through this mechanism model introduced the physical knowledge on the milling process while enhancing the data size to make the learning of the prediction model more reasonable and adequate.

(2) The spatial and temporal features were extracted from the input time series data via convolutional neural networks and gated recurrent units. To enhance the correlation between the sequence data, a bidirectional GRU was introduced to combine the acquired forward and reverse output features into an output feature map. In addition, a multi-headed self-attention mechanism was introduced to aggregate the dimensional weights of the extracted output features in the bidirectional GRU layer to enhance the correlation between the data.

(3) Physical knowledge was introduced into the training phase and loss function part of deep learning to guide the training process of the model. The physically guided loss function can help the model to converge the learning route to the region in the function space that conforms to physical laws, which effectively improves the training efficiency and physical rationality of the model.

**Figure 10:**
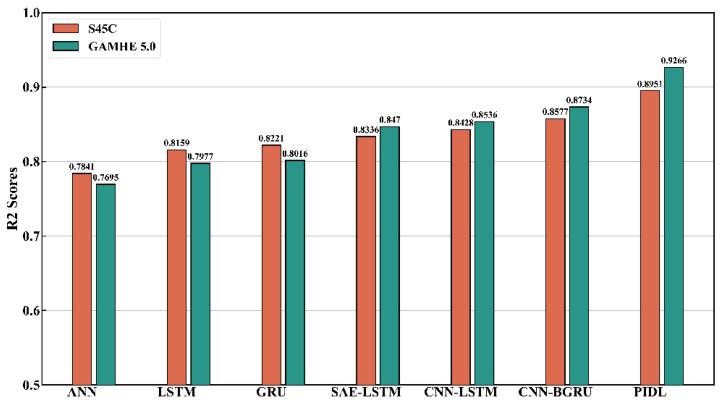
Comparison of R2 metrics under test sets on S45C dataset and reduced-scale GAMHE 5.0 dataset across the methods.

Time series segments of the same size as S45C were cropped from the GAMHE 5.0 dataset to verify the correlation between model effects and dataset size, as well as to check the stability of the proposed model with different data sizes. Surface roughness prediction experiments were conducted on the reduced-scale GAMHE 5.0 dataset, and a comparison of R2 metric values for each model on the test set is shown in Figure 10. It is clear that the prediction accuracy of each model is largely consistent with the results on S45C when the size of the dataset has been reduced. The GRU performed better than the LSTM, further demonstrating its advantage in the case of limited data. The proposed model still has the best performance on the reduced-scale GAMHE 5.0 dataset. CNNs and GRUs are powerful in joint extraction of shallow and deep features of time series, and the physical knowledge also enhances the learning efficiency.

### 4.4. Ablation Experiment

To investigate the effect of different components in the proposed model, the following ablation experiments were performed, where “-” indicates the removal of the current component. “Origin” denotes the complete model proposed in this paper. “-Augmentation” denotes the removal of the data augmentation based on the physical mechanism model. “-CNN” denotes the removal of the feature extraction module based on a CNN. “-Bidirectional” denotes the removal of the bidirectional mechanism of the gated recurrent units. “-Multi-headed” denotes the removal of the multi-headed self-attentive mechanism. “-Loss” denotes the removal of the physical guided loss function. “-Physical” denotes the removal of all physical knowledge introduced in the prediction model, specifically data augmentation and physically guided learning. The proposed model was used on two open-source datasets after removing different components, and a comparison of the model effects on the test set is shown in Table 6.

Through the above ablation experiments, it can be seen that each component of the proposed model contributes to prediction, and the removal of any component will lead to a decrease in the prediction accuracy. Specifically, the prediction accuracy of the model decreased the most for both datasets when the CNN or all physical knowledge introduced into the model was removed. The proposed method performed milling feature extraction by the excellent feature extraction capability of the CNN on time-series data to track the spatial pattern variation caused by the process parameter differences. In terms of the introduction of physical knowledge, it was specifically manifested in data augmentation based on physical mechanism models and physically guided loss functions. Data augmentation enabled the introduction of physical knowledge embedded in the milling process while enhancing the data size, allowing for more adequate learning of the predictive model and improving the generalization of the model. The physically guided loss function can help the model to converge the learning route to the region in the function space that conforms to physical laws, which effectively improves the training efficiency and physical rationality of the model. Then, the performance of the model degrades to some extent both when the data augmentation based on the physical mechanism model and the physically guided loss function were dropped separately. Finally, when the microscopic component of the gating recurrent units was dropped, the model performance slightly degraded. Concretely, the multi-headed component can sufficiently extract correlations between vibrational data dimensions to enhance the connections between them. The bidirectional component can extract the position features of the input data along the forward and reverse dimensions, effectively improving the prediction accuracy. In summary, the removal of the above six components individually in turn all resulted in different degrees of degradation in the model performance. In particular, the absence of the introduced physical knowledge resulted in a clear decrease in accuracy. The reason for the reduction in the training efficiency and physical reasonability is that the model has lost the ability to converge the learning route to a physically lawful region of the function space. Therefore, each component of the model contributes to the model performance.

## 5. Discussion

Through validation experiments on two open-source datasets, the proposed surface roughness prediction model based on deep learning of physical information has better performance compared to comparison models. The experiments demonstrate that convolutional neural networks possess stronger feature extraction capabilities than stacked autoencoders. At the same time, the bidirectional gated recurrent unit can improve the efficiency and learn patterns and connections embedded in time series data for both forward and reverse sequences more comprehensively. The values of both the RMSE and the MAPE increase from the training phase to the testing phase, while the opposite occurs for R2, indicating that the model may be underfitted or overfitted. This is primarily due to the weak generalization ability of the model, resulting from the small training sample. This paper mitigated the overfitting problem of the model by a 10-fold cross-validation. The data augmentation with the constructed physical mechanism model introduced physical knowledge of the milling process while augmenting the amount of data to make the learning of the prediction model more reasonable and adequate. Then, spatial and temporal features were extracted from the input time series data by a convolutional neural network and a gated recurrent unit. To enhance the correlation between sequence data, a bidirectional GRU and a multi-headed self-attention mechanism were applied. Physical knowledge was introduced into the training phase and the loss function part of deep learning to guide the training process of the model, leading the model to converge the learning route to the region in the function space that conforms to physical laws, effectively improving the training efficiency and physical rationality. In the future, this work will further explore surface roughness prediction in online scenarios to ensure the applicability of the proposed model to most manufacturing scenarios such as industrial, medical, and military scenarios.

## 6. Conclusions

Surface roughness has a serious impact on surface hardness, wear resistance and fatigue strength of the product and is a key evaluation index for evaluating the quality of mechanical products, making it significant to study the prediction and optimization of surface roughness. The convergence of machine learning methods to local minima may lead to poor model generalization or results that violate existing physical laws. Therefore, this paper combined physical knowledge with deep learning to propose a physics-guided surface roughness prediction model. This model introduced physical knowledge before and during the training of deep learning. Before training, data augmentation was performed on the limited experimental data by constructing a surface roughness mechanism model with tolerable accuracy. During training, a physically guided loss function was constructed to guide the training process of the model with physical knowledge. Considering the excellent feature extraction capability of convolutional neural networks and gated recurrent units in spatial and temporal dimensions, a CNN–GRU model was adopted as the main prediction model for surface roughness. In addition, the bidirectional GRU and multi-headed self-attention mechanism were introduced to enhance the data intercorrelation. Surface roughness prediction experiments were conducted on the open source S45C steel milling dataset and the micromechanical milling dataset in the GAMHE 5.0 test line. Through a comparison with the prediction results of the latest methods, the proposed model has the highest prediction accuracy on both open source datasets, with the mean absolute percentage error on the test set reduced by 3.029%. In addition, ablation experiments were also conducted on different components to verify the validity of the proposed model and its components.

In the future, our research interests lie in investigating how to achieve precise and efficient optimization of the machining parameters in order to greatly improve the product quality and production efficiency while reducing costs. Moreover, although the proposed prediction model has the highest prediction accuracy on two open-source datasets, it did not directly consider tool wear as a model parameter, but indirectly characterized it through the material removal rate. Therefore, an exploration of the direct relationship between tool wear and surface roughness predictions will be carried out in a follow-up work. The prediction model proposed in this paper is temporarily applicable to surface roughness prediction in offline scenarios where online real-time inspection tasks are not possible. Therefore, the direction of future research lies in how to implement online inspection techniques in this framework. This study performed experimental validation using two existing open-source datasets. Subsequent milling experiments will be conducted and data will be collected personally, and whether there is a correlation between the selection of cutting parameters and surface roughness predictions will be explored within the course of the study. The application of physical knowledge to problems related to overfitting and underfitting will also be of interest.

## Figures and Tables

**Table 1:** Specifications of the tungsten carbide milling cutter.

Cutter Parameter	Setup Value
Diameter of cutter blade	10 mm
Length of blade	30 mm
Total length of cutter	75 mm
Diameter of cutter hilter	10 mm
Number of blades	4
Helix angle	35°

**Table 2:** Cutting parameters and expected material removal rate.

Machining Parameter	Setup Value
Spindle speed (rpm)	900, 1000, 1800, 1900, 2000, 2100, 2700, 3000
Feed rate (mm/min)	228, 240, 252, 320, 400, 420, 532, 560, 588
Feed per tooth (mm/tooth)	0.02–0.09 (total 10 levels)
Cutting depth (mm)	0.5, 0.6, 0.7, 0.8, 0.9, 1
Clamping torque of vise (N-m)	18, 30, 75
MRR per cutter (mm^3^)	Estimated value 0–74.8

**Table 3:** Cutting parameters in the GAMHE 5.0 dataset.

Machining Parameter	Setup Value
Spindle speed (rpm)	30,000, 35,000, 40,000, 45,000
Feed rate (mm/min)	80, 100, 120, 160, 200, 240, 300, 360
Radial depth of cut (mm)	0.15, 0.25, 0.4, 0.5
Axial depth of cut (mm)	0.03, 0.05, 0.08, 0.1
Milling tool radius (mm)	0.15, 0.25, 0.4, 0.5

**Table 4:** Comparison of predictive model metrics on the S45C dataset.

Model	Training Phase			Testing Phase		
	RMSE	MAPE	R2	RMSE	MAPE	R2
ANN [17]	0.1202	16.24%	0.8367	0.1643	25.99%	0.7841
LSTM [39]	0.1034	14.00%	0.8790	0.1518	23.54%	0.8159
GRU [40]	0.1001	13.55%	0.8867	0.1492	23.03%	0.8221
SAE–LSTM [21]	0.0935	12.67%	0.9011	0.1443	22.05%	0.8336
CNN–LSTM [41]	0.0863	11.72%	0.9158	0.1402	21.12%	0.8428
CNN–BGRU [42]	0.0386	5.451%	0.9831	0.1334	16.79%	0.8577
PIDL	0.0273	3.102%	0.9916	0.1145	12.33%	0.8951

**Table 5:** Comparison of predictive metrics on the GAMHE 5.0 dataset.

Model	Training Phase			Testing Phase		
	RMSE	MAPE	R2	RMSE	MAPE	R2
ANN [17]	0.2496	2.662%	0.8389	0.2971	4.880%	0.8079
LSTM [39]	0.2053	1.905%	0.8910	0.2435	3.823%	0.8710
GRU [40]	0.2196	2.147%	0.8753	0.2609	4.149%	0.8519
SAE-LSTM [21]	0.1743	1.434%	0.9214	0.2048	3.149%	0.9087
CNN-LSTM [41]	0.1621	1.286%	0.9321	0.1895	2.929%	0.9219
CNN-BGRU [42]	0.1689	1.367%	0.9262	0.1981	3.052%	0.9146
PIDL	0.0465	0.167%	0.9944	0.0921	1.331%	0.9815

**Table 6:** Comparison of performance results of ablation experiments.

Pattern	S45C			GAMHE 5.0		
	RMSE	MAPE	R2	RMSE	MAPE	R2
Origin	0.1145	12.33%	0.8951	0.0921	1.331%	0.9815
-Augmentation	0.1402	21.11%	0.8430	0.2014	3.097%	0.9118
-CNN	0.1559	24.35%	0.8057	0.2379	3.715%	0.8769
-Bidirectional	0.1362	20.05%	0.8518	0.1689	2.626%	0.9379
-Multi-headed	0.1279	17.56%	0.8691	0.1550	2.412%	0.9477
-Loss	0.1449	22.18%	0.8322	0.2210	3.420%	0.8938
-Physical	0.1503	23.25%	0.8193	0.2570	4.078%	0.8562

## Data Availability

The data that support the findings of this study are openly available at https://drive.google.com/drive/folders/1M6dgDNhHbp8iFmNXeV9HrL6x6AvSshx?usp=share_link (accessed on 15 May 2023).
